# Changes in the Physicochemical Properties of Piperine/*β*-Cyclodextrin due to the Formation of Inclusion Complexes

**DOI:** 10.1155/2016/8723139

**Published:** 2016-02-22

**Authors:** Toshinari Ezawa, Yutaka Inoue, Sujimon Tunvichien, Rina Suzuki, Ikuo Kanamoto

**Affiliations:** ^1^Laboratory of Drug Safety Management, Faculty of Pharmaceutical Sciences, Josai University, 1-1 Keyakidai, Sakado-shi, Saitama 3500295, Japan; ^2^Faculty of Pharmacy, Srinakharinwirot University, Nakhon Nayok 26120, Thailand

## Abstract

Piperine (PP) is a pungent component in black pepper that possesses useful biological activities; however it is practically insoluble in water. The aim of the current study was to prepare a coground mixture (GM) of PP and *β*-cyclodextrin (*β*CD) (molar ratio of PP/*β*CD = 1/1) and subsequently evaluate the solubility of PP and physicochemical properties of the GM. DSC thermal behavior of the GM showed the absence of melting peak of piperine. PXRD profile of the GM exhibited halo pattern and no characteristic peaks due to PP and *β*CD were observed. Based on Job's plot, the PP/*β*CD complex in solution had a stoichiometric ratio of 1/1. Raman spectrum of the GM revealed scattering peaks assigned for the benzene ring (C=C), the methylene groups (CH_2_), and ether groups (C-O-C) of PP that were broaden and shifted to lower frequencies. SEM micrographs showed that particles in the GM were agglomerated and had rough surface, unlike pure PP and pure *β*CD particles. At 15 min of dissolution testing, the amount dissolved of PP in the GM was dramatically increased (about 16 times) compared to that of pure PP. Moreover the interaction between PP and *β*CD cavity was detected by ^1^H-^1^H NMR nuclear Overhauser effect spectroscopy NMR spectroscopy.

## 1. Introduction

**Figure 1:**
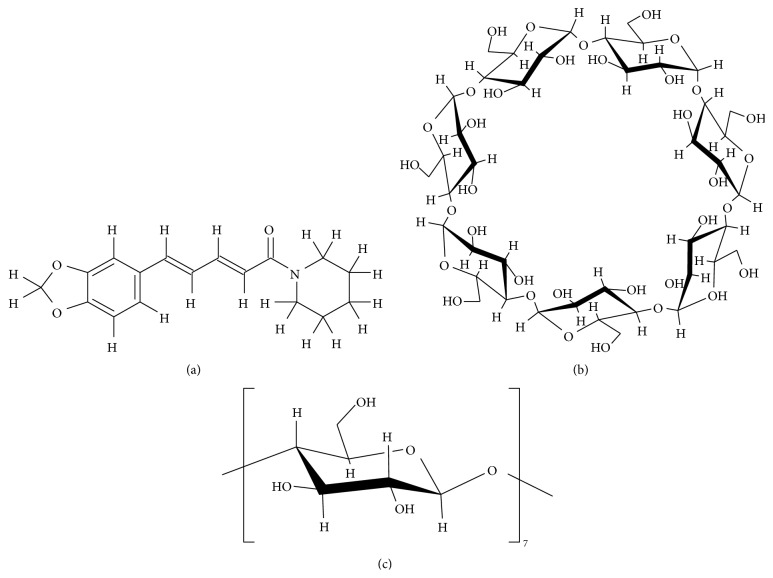
Chemical structure of (a) piperine (PP), (b) *β*-cyclodextrin (*β*CD), and (c) D-glucopyranose.

Piperine [(2E,4E)-1-[5-(1,3-benzodioxol-5yl)-1-oxo-2,4-pentadienyl]piperidine, denoted here as PP] (Figure 1) is a component found in black pepper. PP molecule consists of piperidine and piperic acid linked by an amide bond. PP is pungent; however its* cis*-trans isomers, that is, isopiperine, chavicine, and isochavicine, have little pungency [1]. PP has been reported for its slight insecticidal activity against third instar larvae of* Culex pipiens* pallens,* Aedes aegypti*, and* A. togoi* [2]. PP at a concentration of 100–500 *μ*g/mL has been reported to exhibit antibacterial action against* Staphylococcus aureus*,* Pseudomonas aeruginosa*,* Bacillus subtilis*, and* Escherichia coli* [3]. In addition, PP has been reported to reduce thiobarbituric acid reactive substances and the degree of lipid peroxidation [4]. PP activates the sympathetic nerves through the vagus nerve by acting on TRPV1 receptors present at the endings of sensory nerves and the vagus nerve. It is also reported that PP increases the energy consumption of skeletal muscle and brown adipose tissue [5, 6] and facilitates lipolysis in white adipose tissue [7]. Thus, PP has recently drawn attention as a useful spice for use in functional foods. PP is relatively stable in black pepper; however, it is readily isomerized when exposed to ultraviolet light [8]. Therefore, PP in pepper oleoresin must be shielded from light. In addition, because of its low aqueous solubility, PP is unable to exhibit its beneficial actions properly.

Cyclodextrins (CDs) are cyclic oligosaccharides consisting of D-glucopyranose linked by *α*-1,4 glycosidic bonds. CDs are classified as *α*, *β*, and *γ*CD according to the number of their D-glucopyranose units. CDs have a hydrophilic outer ring and a hydrophobic cavity. CDs are known to form inclusion complexes with various hydrophobic guest molecules by hydrophobic interaction in an aqueous solution [9–11]. Cogrinding is a technique of applying mechanical energy to produce inclusion complexes that is suitable for hydrolysis-prone compounds [12]. The process of encapsulating a guest molecule into the CD cavity has been used in various areas [13]. An inclusion complex of prostaglandin E_2_/*α*-CD with increased solubility of prostaglandin E_2_ has already been utilized in clinical settings. CDs are also widely used as a deodorant and food stabilizer in order to mask undesired properties of active compound [14], to release active compound in a sustained manner [15], and to stabilize active compound [16, 17]. One example is the inclusion complexes between poorly water-soluble capsaicin and *β*-cyclodextrin (*β*CD) that led to enhanced solubility and stability of capsaicin [18]. Similarly, formation of curcumin/hydroxypropyl-*β*CD complexes [19] and alpinetin/hydroxypropyl-*β*CD complexes [20] has been reported. Also recently, improvements in the biological activity of the complex formation between Q10 and CD and the development of functional foods have been made. Therefore, applications to the food and pharmaceutical sectors have been increasingly expected [21]. These findings are expected to lead to greater clinical use of drugs complexed with a CD in the fields of health care products and herbal medicines. PP can promote the absorption of *β*-carotene (vitamin A) and vitamin B6 by interfering the substance removal process from cells by the “pump” protein p-glycoprotein. PP has also been reported to facilitate the absorption of the active components of healthy food products such as turmeric and coenzyme Q10 [22]. The application to the food field is expected for the pharmaceutical research complexation technique of medical supplies. Improvement of the solubility and stability of PP will lead to greater use of PP as a health supplement in functional food in the future. Therefore, the aims of this study were to prepare inclusion complexes of PP and *β*CD by cogrinding method and to evaluate the dissolution behavior of PP and the physical-chemical properties of the inclusion complexes.

## 2. Materials and Methods

### 2.1. Materials

Piperine (PP: 98% for biochemistry) was purchased from Wako Pure Chemical Co., Ltd. *β*CD was donated by Cyclo Chem Co., Ltd. (Tokyo, Japan) and was used after storing at a temperature of 40°C and relative humidity of 82% for 7 days. The humidity was controlled in order to prepare stable *β*-cyclodextrin 10.5 hydrate. The humidity was controlled, in order to perform weight measurement after unifying the moisture content of cyclodextrin. HPLC solvents of HPLC grade were purchased from Wako Pure Chemical Co., Ltd. All other chemicals and solvents were of analytical grade.

### 2.2. Methods

#### 2.2.1. Preparation of a Physical Mixture and Ground Mixtures

A physical mixture (PM) of PP/*β*CD at a molar ratio of 1/1 was prepared by blending the two compounds for 1 minute using a vortex mixer (Model TM-151, IWAKI GLASS Co.). Ground mixtures (GMs) of PP/*β*CD at a molar ratios of 2/1, 1/1, and 1/2 by grinding the PMs (1.0 g) were prepared by cogrinding the two compounds for 60 min using a vibration rod mill (TI-500ET, CMT Co.) with an aluminum cell.

#### 2.2.2. Differential Scanning Calorimetry (DSC)

Thermal behavior of the samples was analyzed using a differential scanning calorimeter (Thermo plus Evo, Rigaku) under nitrogen flow rate of 60 mL/min. The samples were heated at a scanning rate of 5.0°C/min from 30 to 150°C.

#### 2.2.3. Powder X-Ray Diffraction (PXRD)

Powder X-ray diffraction was performed using an X-ray diffractometer (MiniFlex II, Rigaku) with Cu Ka radiation, a voltage of 30 kV, a current of 15 mA, a scan range of 3–35°, and a scan rate of 4°/min. The intensities were measured with NaI scintillation counter coupled to a discriminator.

#### 2.2.4. Determination of the Complexation Stoichiometry

The stoichiometry of the complex formation between PP and *β*CD was determined using Job's method with continuous variation [23]. To maintain a constant total concentration of PP and *β*CD, the comparison ratio of the guest molecules was varied between 0 and 1 in the test solution. After the test solution reached an equilibrium state, samples were filtrated through a 0.45-*μ*m membrane filter. The PP amount in the sample solutions was measured using a UV-2500PC ultraviolet-visible spectrophotometer (manufactured by Shimadzu Corporation) at a wavelength of *λ* = 341 nm (measurement is *n* = 3).

#### 2.2.5. Raman Spectroscopy

Raman spectra of the samples were recorded using a spectrometer (Cart-Mountable RamanRxn2^TM^ Analyzer-1000 nm, KAISER). The measurement condition is as follows: a scan range of 200–1700 cm^−1^, a spectral resolution of 5 cm^−1^, an f/1.8 imaging spectrograph with a holographic transmission grating, and a detector (TE-cooled 1024-array detector).

#### 2.2.6. Scanning Electron Microscopy (SEM)

SEM was performed using a S3000N Scanning Electron Microscope (Hitachi High-Technologies Corporation) at an acceleration voltage of 15 kV. Samples were mounted on aluminum SEM stubs. These sample stubs were coated with a thin layer of gold to make them electrically conductive.

#### 2.2.7. ^1^H-^1^H Nuclear Overhauser Effect Spectroscopy (NOESY) NMR Spectroscopy

Two-dimensional (2D) NOESY NMR spectroscopy and selective 1D NMR spectroscopy were performed using an NMR spectrometer (Varian NMR System 700NB, Agilent) with a cold probe operating at 699.7 MHz and a D_2_O solution. The measurement conditions were as follows: an acquisition time of 7.0 *μ*s, a pulse width of 90°, a relaxation delay of 0.267 s, a mixing time of 4.500 s, a fixed delay of 1.500 s, and a temperature of 298 K.

#### 2.2.8. Dissolution Testing

Dissolution testing was conducted in accordance with the paddle method in the 16th edition of the Japanese Pharmacopoeia. Dissolution experiment was performed using a dissolution apparatus (NTR-593, Toyama Sangyo) at 37 ± 0.5°C with 900 mL of distilled water that was stirred with paddle at 50 rpm. Each 10 mL of a dissolution sample was collected at 0, 5, 10, 15, 30, and 60 min, respectively. Sample solutions were filtered through a 0.45-*μ*m membrane filters and diluted with a water/methanol in a ratio of 1/1 as appropriate. The amount of PP in diluted sample solutions was measured using a UV-2500PC ultraviolet-visible spectrophotometer (manufactured by Shimadzu Corporation) at a wavelength of *λ* = 345 nm. Measurement is *N* = 3. Apparent dissolution profile was at sink conditions.

## 3. Results and Discussion

### 3.1. Examination of Thermal Behavior

**Figure 2:**
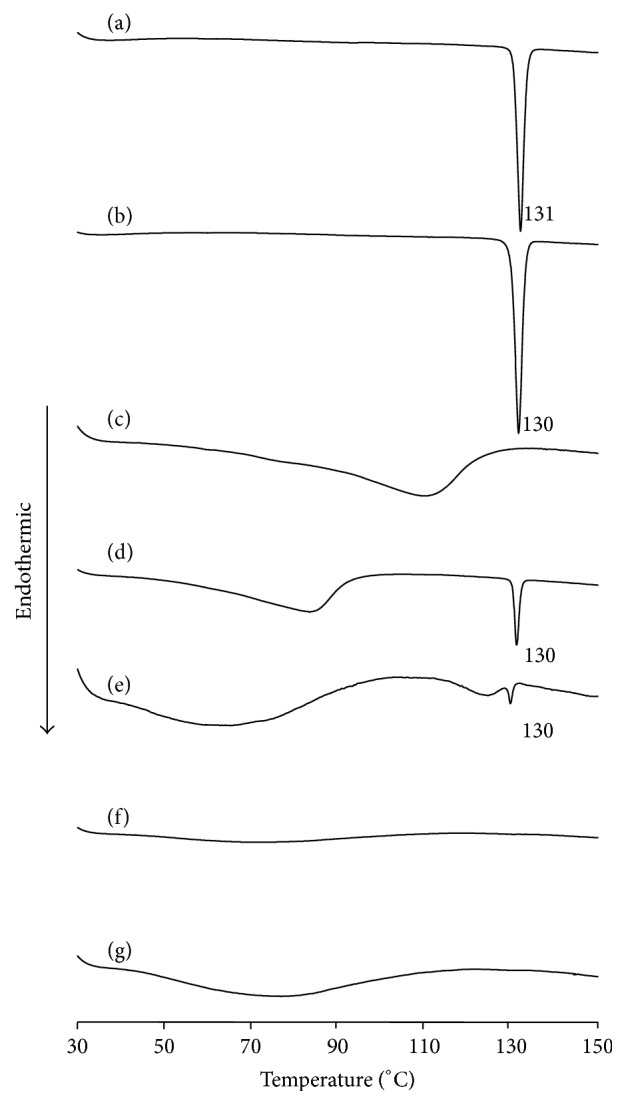
DSC curves of (a) PP, (b) PP ground for 60 min, (c) *β*CD, (d) PM (PP/*β*CD = 1/1), (e) GM (PP/*β*CD = 2/1), (f) GM (PP/*β*CD = 1/1), and (g) GM (PP/*β*CD = 1/2).

DSC was performed to examine the thermal properties of PP and *β*CD in mixtures (Figure 2). The thermograms of intact PP, PP ground for 60 min, the PM (PP/*β*CD = 1/1), and the GM (PP/*β*CD = 2/1) showed an endothermic peak due to the melting of PP at around 130°C (Figure 2(a, b, d, e)), whereas it was not observed in the thermograms of GM (PP/*β*CD = 1/1) and GM (PP/*β*CD = 1/2) (Figure 2(f, g)). According to a study by Giordano et al., when CD and a drug had molecular interaction the melting point of the drug disappeared in DSC [24, 25]. Thus, the current results similarly revealed that GM (PP/*β*CD = 1/1 or PP/*β*CD = 1/2) lacked a peak due to melting of PP, so some form of molecular interaction between PP and *β*CD is presumed to have occurred. Intact *β*CD's thermogram exhibited a broad endothermic peak at around 100°C which arising from the dehydration. The GM (PP/*β*CD = 1/2) also showed smaller endothermic peak due to water loss (Figure 2(g)). This was presumably the result of a water loss from *β*CD which was not involved in inclusion. Thermal analysis exhibited changes in thermal behavior, and the findings above suggest molecular interaction between coground PP and *β*CD and changes in the properties of PP. DSC results suggested that PP/*β*CD complexes might be produced at a molar ratio of 1/1 or 1/2 PP/*β*CD.

### 3.2. Examination of the Crystalline State

**Figure 3:**
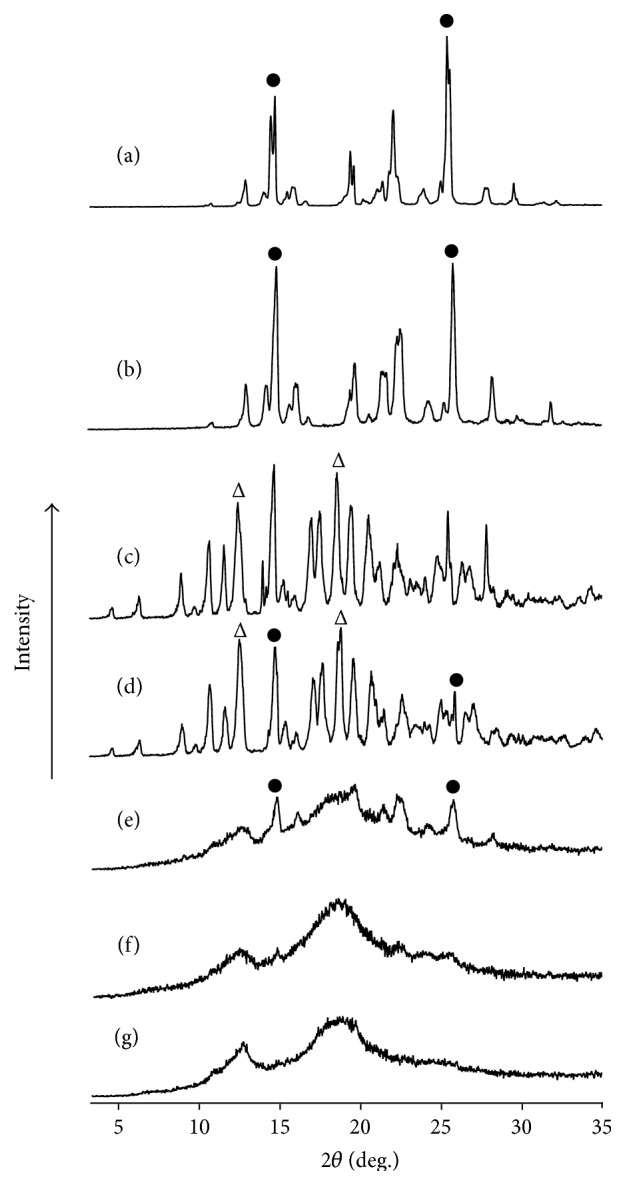
PXRD patterns of (a) PP, (b) PP ground for 60 min, (c) *β*CD, (d) PM (PP/*β*CD = 1/1), (e) GM (PP/*β*CD = 2/1), (f) GM (PP/*β*CD = 1/1), and (g) GM (PP/*β*CD = 1/2). ●: PP original, Δ: *β*CD original.

PXRD was performed to examine the crystalline state of PP and *β*-CD in the mixtures (Figure 3). Intact PP exhibited characteristic diffraction peaks (indicated by ●) at 2*θ* = 12.9° and 19.6°, respectively, whereas *β*CD showed characteristic diffraction peaks (indicated by Δ) at 2*θ* = 12.6° and 17.8°, respectively (Figure 3(a, b, c)). Ground PP showed a similar PXRD pattern to that of intact PP, indicating that no phase transition occurred. The PM (PP/*β*CD = 1/1) exhibited superimposed PXRD patterns of PP and *β*CD crystals (Figure 3(d)). The GM (PP/*β*CD = 2/1) had decreased crystallinity compared to the PM and the small diffraction peaks due to PP were observed in the profile. However the GM (PP/*β*CD = 1/1) and GM (PP/*β*CD = 1/2) samples showed halo PXRD patterns (Figure 3(f, g)), indicating amorphous formation of PP [26, 27]. Thus, mechanical energy in the form of distortion or percussion might induce the mechanochemical interaction between PP and *β*CD in a solid state.

### 3.3. Analysis of the Complexation Stoichiometry

**Figure 4:**
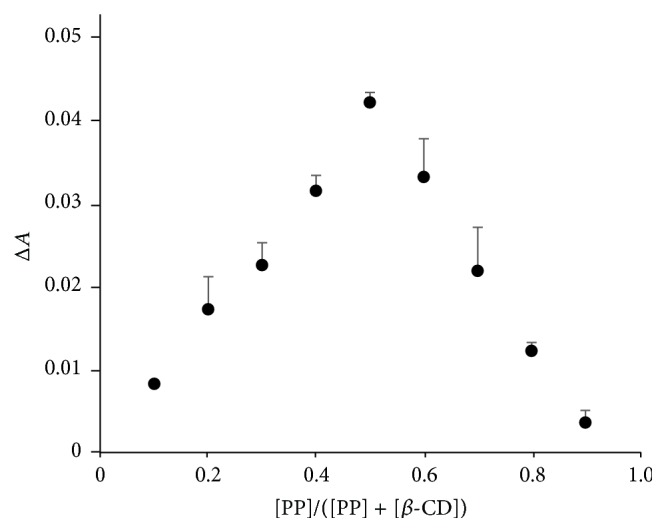
Job's plot of the PP/*β*CD system. Results were expressed as mean ± SE (*n* = 3).

Jadhav and Vavia determined the molar ratio of *β*CD and danazol complex based on Job's plot [28]. Continuous changes in absorbance (Job's plot) in relation with the proportion of PP and *β*CD were used to calculate the proportion of the two compounds in complexes (Figure 4). The maximum absorbance was obtained when the PP/([PP] + [*β*CD]) was 0.5. Thus, it was explained that PP and *β*CD formed complexes at a molar ratio of 1/1.

### 3.4. Examination of Molecular Interaction in a Solid State

**Figure 5:**
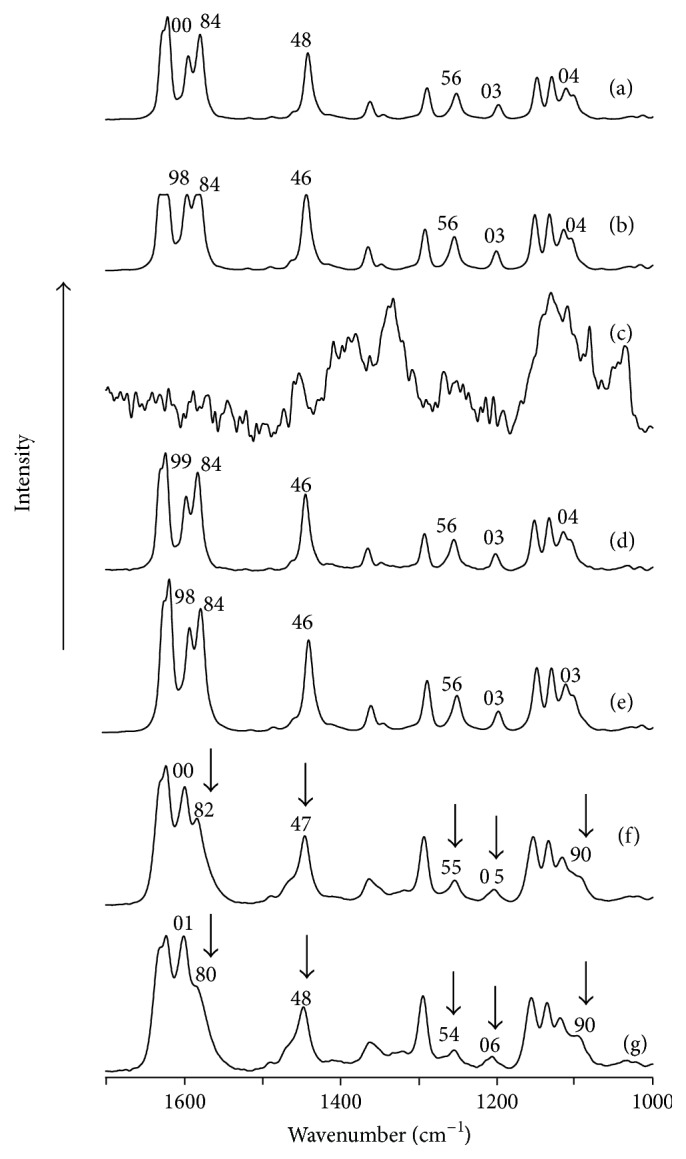
Raman spectra of (a) PP, (b) PP ground for 60 min, (c) *β*CD, (d) PM (PP/*β*CD = 1/1), (e) GM (PP/*β*CD = 2/1), (f) GM (PP/*β*CD = 1/1), and (g) GM (PP/*β*CD = 1/2).

Results of DSC, PXRD, and Job's plot suggested that PP and *β*CD formed complexes at a molar ratio of 1/1. Raman spectroscopy was performed to investigate the molecular state of the PP and *β*CD complex in a solid state (Figure 5). Raman spectroscopy is more suitable for investigating an interaction of symmetrically substituted C=C than infrared spectroscopy. It can be used to ascertain the degree of crystallization and stress on a crystal lattice as well [29]. For pure PP, its C=C of the aromatic ring, CH of the phenyl group, CH_2_, and C-O-C produced scattering peaks at 1584, 1104, 1203, and 1256 cm^−1^, respectively. Scattering peaks due to PP were also observed in the PM's spectrum at the same wave numbers (Figure 5(d)). For the GM (PP/*β*CD = 1/1), broaden scattering peaks assigned for C=C of the aromatic ring, CH of the phenyl group, CH_2_, and C-O-C were recorded at 1582, 1090, 1205, and 1255 cm^−1^, respectively. These peaks were shifted to lower frequencies in comparison to those of pure PP (Figure 5(f)). Broaden and shifted scattering peaks of the GM (PP/*β*CD = 1/1) might be due to the fact that molecular vibrational relaxation time is inversely proportional to an increase in the half-width of the peak. When PP molecule was included in the CD cavity, the interaction between the two compounds may suppress symmetrical stretching completely, resulting in broaden and shifted scattering peaks. This suggested that C=C of the aromatic ring, CH of the phenyl group, CH_2_, and C-O-C functional groups of the PP molecule were involved in complex formation with *β*CD.

### 3.5. Examination of Morphological Properties

**Figure 6:**
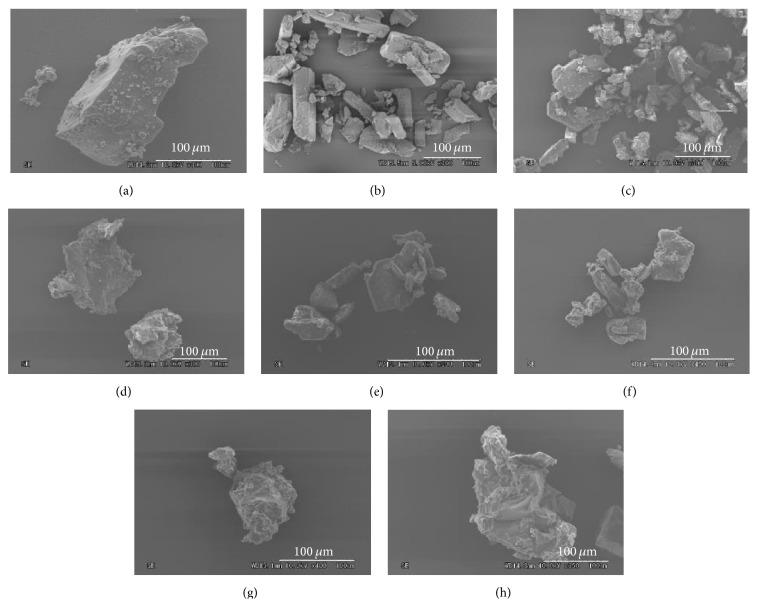
SEM micrographs of (a) PP, (b) PP ground for 60 min, (c) *β*-CD, (d) *β*-CD ground for 60 min, (e) PM (PP/*β*CD = 1/1), (f) GM (PP/*β*CD = 1/2), (g) GM (PP/*β*CD = 1/1), and (h) GM (PP/*β*CD = 1/2).

SEM micrographs of samples are depicted in Figure 6. The surface of PP particle was rather smooth and its size was about 200 *μ*m (Figure 6(a)). After grinding for 60 min, the PP particles had smaller size (50 *μ*m in size) and rougher surface and some particles were agglomerated (Figure 6(b)). Also, *β*CD particles had smooth surface with size of 100 *μ*m (Figure 6(c)). Particles of intact PP and *β*-CD were in block shape. There was no marked particle surface change observed in the PM (Figure 6(e)).

The GM (PP/*β*CD = 1/1) contained rough-surface particles of varied sizes (about 100 *μ*m); some particles were aggregated (Figure 6(g)). Mechanical energy arising from grinding process might presumably affect the particle surface, leading to particle size reduction. The particles of the GMs clearly differed from that of pure PP; they had rough surface and were agglomerated. Differences in particle surface (i.e., rougher surface) and particle agglomeration have been reported when *β*CD encapsulates a drug molecule [30]. Thus, PP molecules and *β*CD molecules are likely to form inclusion complexes in a solid state.

### 3.6. Assessment of Dissolution

Results mentioned above suggested that PP and *β*CD formed complexes via cogrinding process. Dissolution testing was conducted to monitor changes in the aqueous solubility of PP as a consequence of the inclusion complexation.

**Figure 7:**
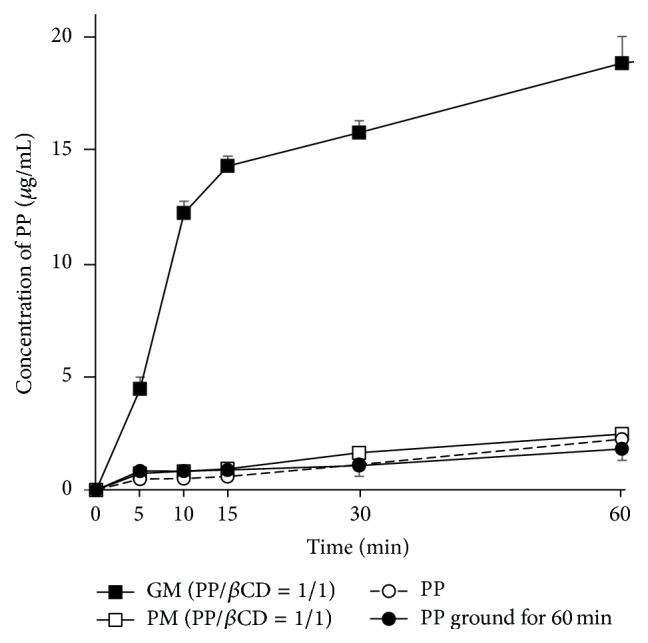
Dissolution profiles of PP/*β*CD systems (37 ± 0.5°C). Results are presented as the mean ± SD (*n* = 3).

Dissolution profiles of intact PP, PP ground for 60 min, a PM (PP/*β*CD = 1/1), and a GM (PP/*β*CD = 1/1) are shown in Figure 7. The amounts dissolved of PP after 15 min from intact PP, PP ground for 60 min, a PM (PP/*β*CD = 1/1), and a GM (PP/*β*CD = 1/1) samples were 0.88 *μ*g/mL, 0.59 *μ*g/mL, 1.0 *μ*g/mL, and 14.37 *μ*g/mL, respectively. The GM (PP/*β*CD = 1/1) exhibited a remarkably improved dissolution compared to other samples. PP ground for 60 min did not have changes in solubility compared to PP alone. Thus, the increased surface area of particles due to cogrinding did not improve the solubility of the GM (PP/*β*CD = 1/1). Due to the solid-state interaction of *β*CD molecule with the aromatic ring of PP molecule which was confirmed by Raman spectra, solubility of PP was enhanced dramatically.

### 3.7. Examination of Molecular States in Solution

**Figure 8:**
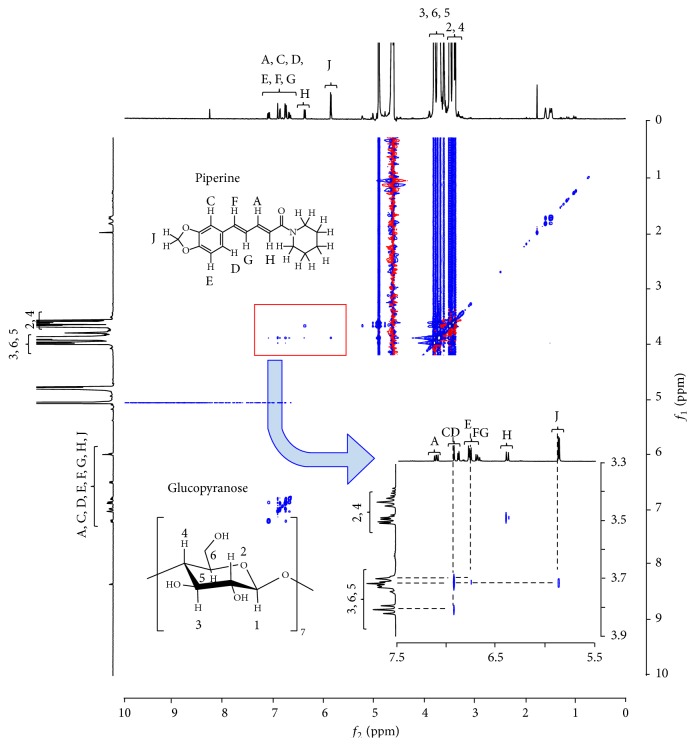
^1^H-^1^H NOESY NMR spectrum produced by the GM (molar ratio of PP/*β*-CD = 1/1) in D_2_O. *X* is 5.7–7.5 and *Y* is 3.3–3.9.

**Figure 9:**
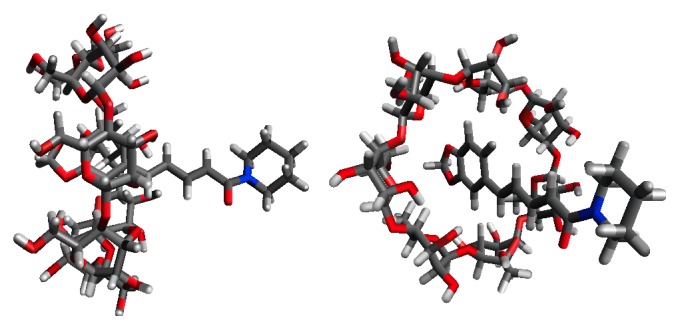
Structural view of a PP/*β*CD complex.

Owing to an improved solubility of the GM (PP/*β*CD = 1/1), two-dimensional NMR spectroscopy was performed to confirm the existence of interaction between PP and *β*-CD in solution and to determine the form of inclusion. ^1^H-^1^H nuclear Overhauser effect spectroscopy (NOESY) NMR spectroscopy is typically used to estimate the relative positions of protons in the CD cavity and protons of the guest molecule for analyzing the structure of inclusion complex [31]. The ^1^H-^1^H NOESY NMR spectrum of the GM (PP/*β*CD = 1/1) is illustrated in Figure 8. Cross peaks were observed between the peaks for H-C, H-D, and H-E of the benzene ring of PP and H-3 and H-5 of *β*CD. An interesting finding is that cross peaks were also observed between H-J of an ester group of PP and H-6 of *β*CD. This could be interpreted by the fact that protons of the benzene ring and ester groups of PP interacted with protons of the cavity of *β*CD. This could be explained by the fact that a portion of PP molecule, that is, from an ester group to the benzene ring, was included in the vast cavity of CD (Figure 9). This evidence also confirms that the formation of inclusion complexes between PP and *β*CD helped improve the solubility of PP.

## 4. Conclusion

Inclusion complex of PP and *β*CD was produced by cogrinding process and their physicochemical properties were investigated by means of PXRD, DSC, Raman spectroscopy, and SEM, respectively. Changes in the solid-state physicochemical properties, particle shape, and particle surface of the GM at a molar ratio of 1/1 were detected. Job's plot confirmed that PP and *β*CD at a molar ratio of 1/1 formed inclusion complexes in solution. The solubility of PP was increased as a result of inclusion in *β*CD. In addition, ^1^H-^1^H NOESY NMR spectra revealed the form in which PP was included in *β*CD. The increased solubility of PP was attributed to the interaction between the aromatic ring of PP (which is lipophilic) and the cavity of *β*CD. This observation indicates that PP can be used beneficially in the development of health care supplements. The stability of PP on its use in food supplement and the mechanism by which PP is included in *β*CD by different preparation methods need to be thoroughly investigated in the future.

## References

[1] Grewe R., Freist W., Neumann H., Kersten S. (1970). Über die Inhaltsstoffe des schwarzen Pfeffers. *Chemische Berichte*.

[2] Park I.-K., Lee S.-G., Shin S.-C., Park J.-D., Ahn Y.-J. (2002). Larvicidal activity of isobutylamides identified in Piper nigrum fruits against three mosquito species. *Journal of Agricultural and Food Chemistry*.

[3] Karsha P. V., Lakshmi O. B. (2010). Antibacterial activity of black pepper (*Piper nigrum* Linn.) with special reference to its mode of action on bacteria. *Indian Journal of Natural Products and Resources*.

[4] Rauscher F. M., Sanders R. A., Watkins J. B. (2000). Effects of piperine on antioxidant pathways in tissues from normal and streptozotocin-induced diabetic rats. *Journal of Biochemical and Molecular Toxicology*.

[5] Okumura Y., Narukawa M., Iwasaki Y. (2010). Activation of TRPV1 and TRPA1 by black pepper components. *Bioscience, Biotechnology and Biochemistry*.

[6] Jamwal D. S., Singh J. (1993). Effects of piperine on enzyme activities and bioenergetic functions in isolated rat liver mitochondria and hepatocytes. *Journal of Biochemical Toxicology*.

[7] Vijayakumar R. S., Nalini N. (2006). Piperine, an active principle from Piper nigrum, modulates hormonal and apolipoprotein profiles in hyperlipidemic rats. *Journal of Basic and Clinical Physiology and Pharmacology*.

[8] Hashimoto K., Yaoi T., Koshiba H. (1996). Photochemical isomerization of piperine, a pungent constituent in pepper. *Food Science and Technology International*.

[9] Brewster M. E., Loftsson T. (2007). Cyclodextrins as pharmaceutical solubilizers. *Advanced Drug Delivery Reviews*.

[10] Loftsson T., Duchêne D. (2007). Cyclodextrins and their pharmaceutical applications. *International Journal of Pharmaceutics*.

[11] Tao T., Zhao Y., Wu J., Zhou B. (2009). Preparation and evaluation of itraconazole dihydrochloride for the solubility and dissolution rate enhancement. *International Journal of Pharmaceutics*.

[12] Borba P. A., Pinotti M., Andrade G. R. (2015). The effect of mechanical grinding on the formation, crystalline changes and dissolution behaviour of the inclusion complex of telmisartan and *β*-cyclodextrins. *Carbohydrate Polymers*.

[13] Del Valle E. M. M. (2004). Cyclodextrins and their uses: a review. *Process Biochemistry*.

[14] Tan Q., Zhang L., Zhang L., Teng Y., Zhang J. (2012). Design and evaluation of an economic taste-masked dispersible tablet of pyridostigmine bromide, a highly soluble drug with an extremely bitter taste. *Chemical and Pharmaceutical Bulletin*.

[15] Martin A., Tabary N., Leclercq L. (2013). Multilayered textile coating based on a *β*-cyclodextrin polyelectrolyte for the controlled release of drugs. *Carbohydrate Polymers*.

[16] Cappello B., Di Maio C., Iervolino M., Miro A. (2006). Improvement of solubility and stability of valsartan by hydroxypropyl-*β*-cyclodextrin. *Journal of Inclusion Phenomena*.

[17] Szente L., Szejtli J. (2004). Cyclodextrins as food ingredients. *Trends in Food Science & Technology*.

[18] Shen C., Yang X., Wang Y., Zhou J., Chen C. (2012). Complexation of capsaicin with *β*-cyclodextrins to improve pesticide formulations: effect on aqueous solubility, dissolution rate, stability and soil adsorption. *Journal of Inclusion Phenomena and Macrocyclic Chemistry*.

[19] Yallapu M. M., Jaggi M., Chauhan S. C. (2010). *β*-cyclodextrin-curcumin self-assembly enhances curcumin delivery in prostate cancer cells. *Colloids and Surfaces B: Biointerfaces*.

[20] Ma S.-X., Chen W., Yang X.-D. (2012). Alpinetin/hydroxypropyl-*β*-cyclodextrin host-guest system: preparation, characterization, inclusion mode, solubilization and stability. *Journal of Pharmaceutical and Biomedical Analysis*.

[21] Terao K., Nakata D., Fukumi H. (2006). Enhancement of oral bioavailability of coenzyme Q10 by complexation with *γ*-cyclodextrin in healthy adults. *Nutrition Research*.

[22] Badmaev V., Majeed M., Prakash L. (2000). Piperine derived from black pepper increases the plasma levels of coenzyme Q10 following oral supplementation. *The Journal of Nutritional Biochemistry*.

[23] Djedaïni F., Lin S. Z., Perly B., Wouessidjewe D. (1990). High-field nuclear magnetic resonance techniques for the investigation of a *β*-cyclodextrin:indomethacin inclusion complex. *Journal of Pharmaceutical Sciences*.

[24] Giordano F., Novak C., Moyano J. R. (2001). Thermal analysis of cyclodextrins and their inclusion compounds. *Thermochimica Acta*.

[25] Aigner Z., Berkesi O., Farkas G., Szabó-Révész P. (2012). DSC, X-ray and FTIR studies of a gemfibrozil/dimethyl-*β*-cyclodextrin inclusion complex produced by co-grinding. *Journal of Pharmaceutical and Biomedical Analysis*.

[26] Ogawa N., Higashi K., Nagase H. (2010). Effects of cogrinding with *β*-cyclodextrin on the solid state fentanyl. *Journal of Pharmaceutical Sciences*.

[27] Mura P., Bettinetti G. P., Cirri M., Maestrelli F., Sorrenti M., Catenacci L. (2005). Solid-state characterization and dissolution properties of naproxen-arginine-hydroxypropyl-*β*-cyclodextrin ternary system. *European Journal of Pharmaceutics and Biopharmaceutics*.

[28] Jadhav G. S., Vavia P. R. (2008). Physicochemical, in silico and in vivo evaluation of a danazol-*β*-cyclodextrin complex. *International Journal of Pharmaceutics*.

[29] Hédoux A., Paccou L., Guinet Y., Willart J.-F., Descamps M. (2009). Using the low-frequency Raman spectroscopy to analyze the crystallization of amorphous indomethacin. *European Journal of Pharmaceutical Sciences*.

[30] Yallapu M. M., Jaggi M., Chauhan S. C. (2010). *β*-Cyclodextrin-curcumin self-assembly enhances curcumin delivery in prostate cancer cells. *Colloids and Surfaces B: Biointerfaces*.

[31] Pitha J., Pitha J. (1985). Amorphous water-soluble derivatives of cyclodextrins: nontoxic dissolution enhancing excipients. *Journal of Pharmaceutical Sciences*.

